# Supplementary material for: Transcriptome Profiling of Two Camellia japonica Cultivars with Different Heat Tolerance Reveals Heat Stress Response Mechanisms
Source: Plants (Basel). 2024 Nov 2;13(21):3089. doi: 10.3390/plants13213089 (PMC11548091; doi:10.3390/plants13213089)
Supplement: Supplementary file 1 [file plants-13-03089-s001.zip › plants-3235590-supplementary.pdf]

Supplementary material

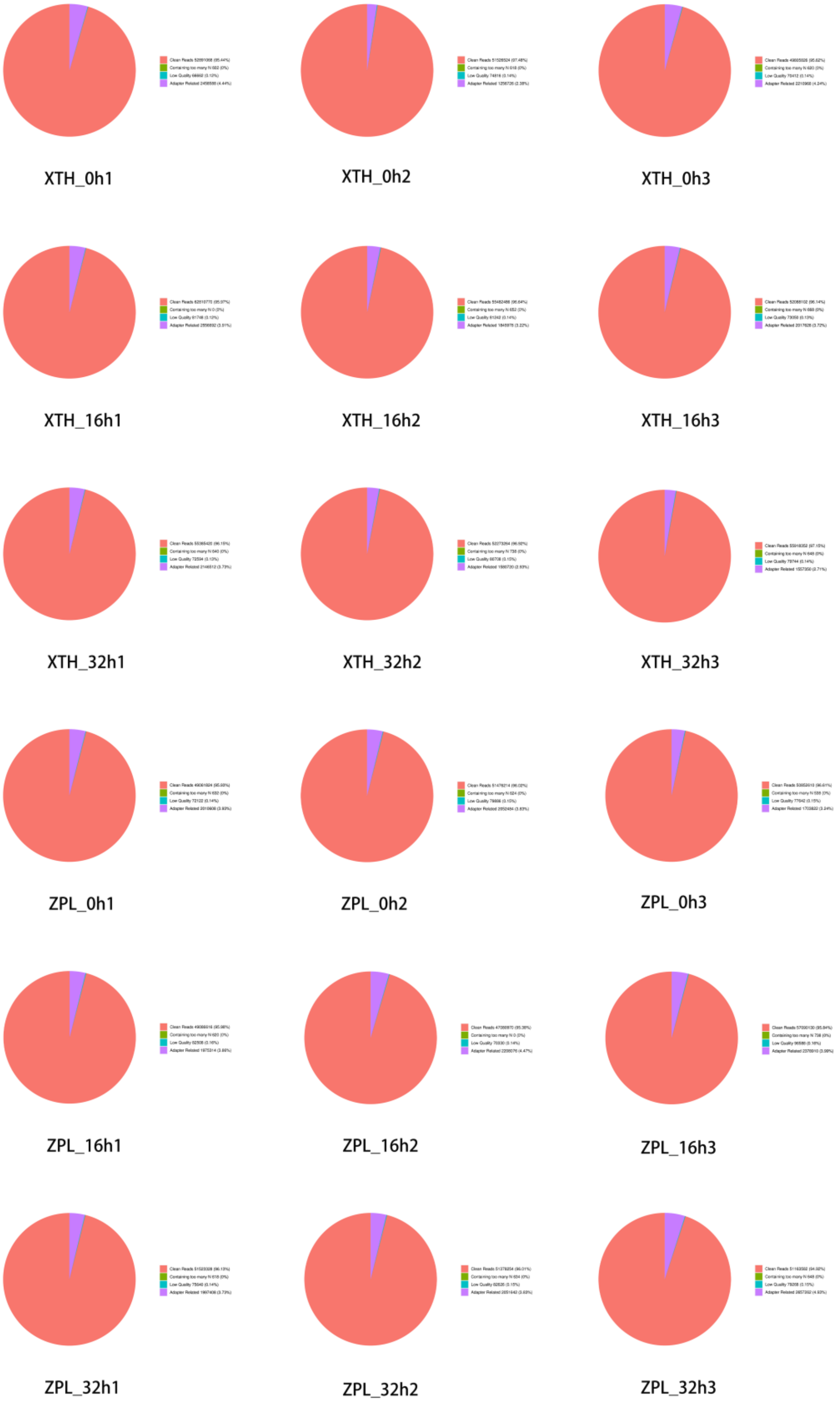

Figure S1. Quality of sequencing readings of 18 RNA sequencing samples

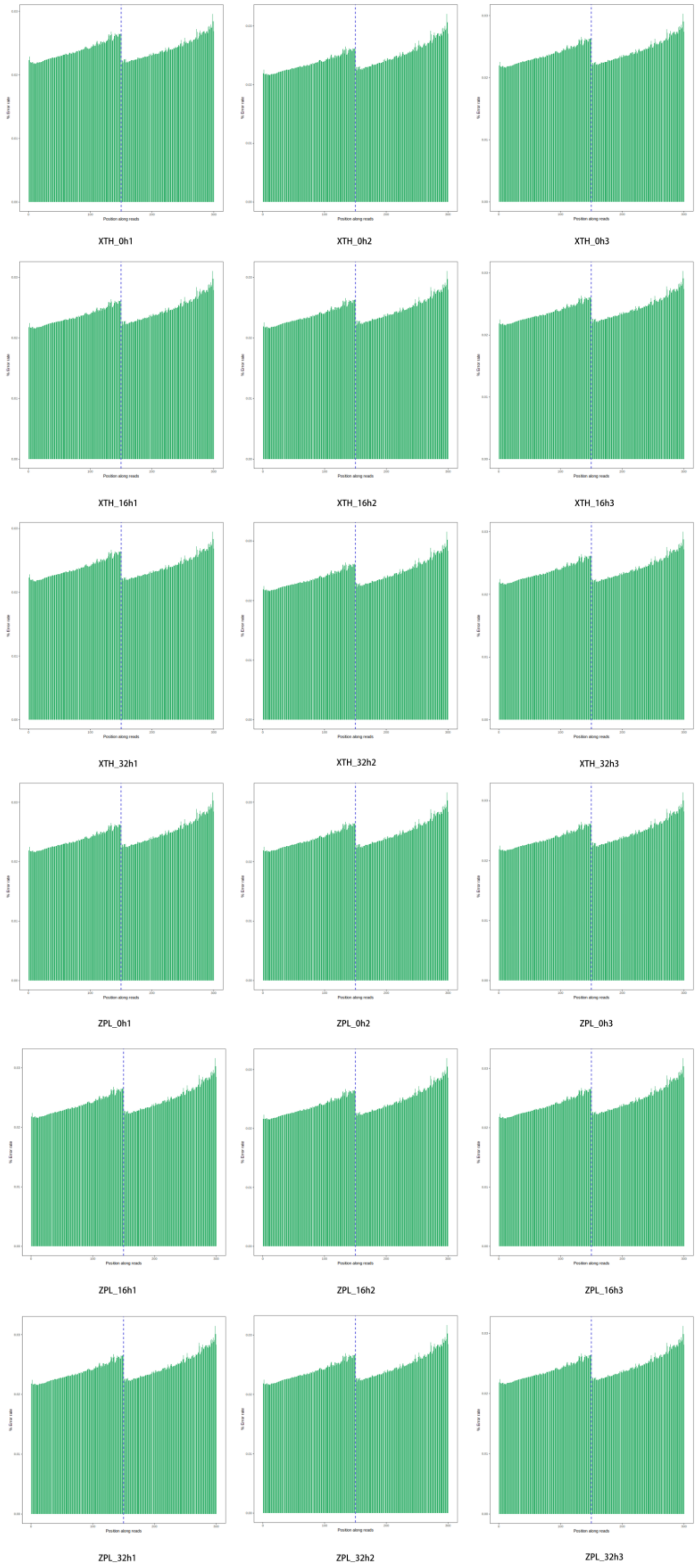

Figure S2. Distribution of sequencing error bases in 18 RNA sequencing samples

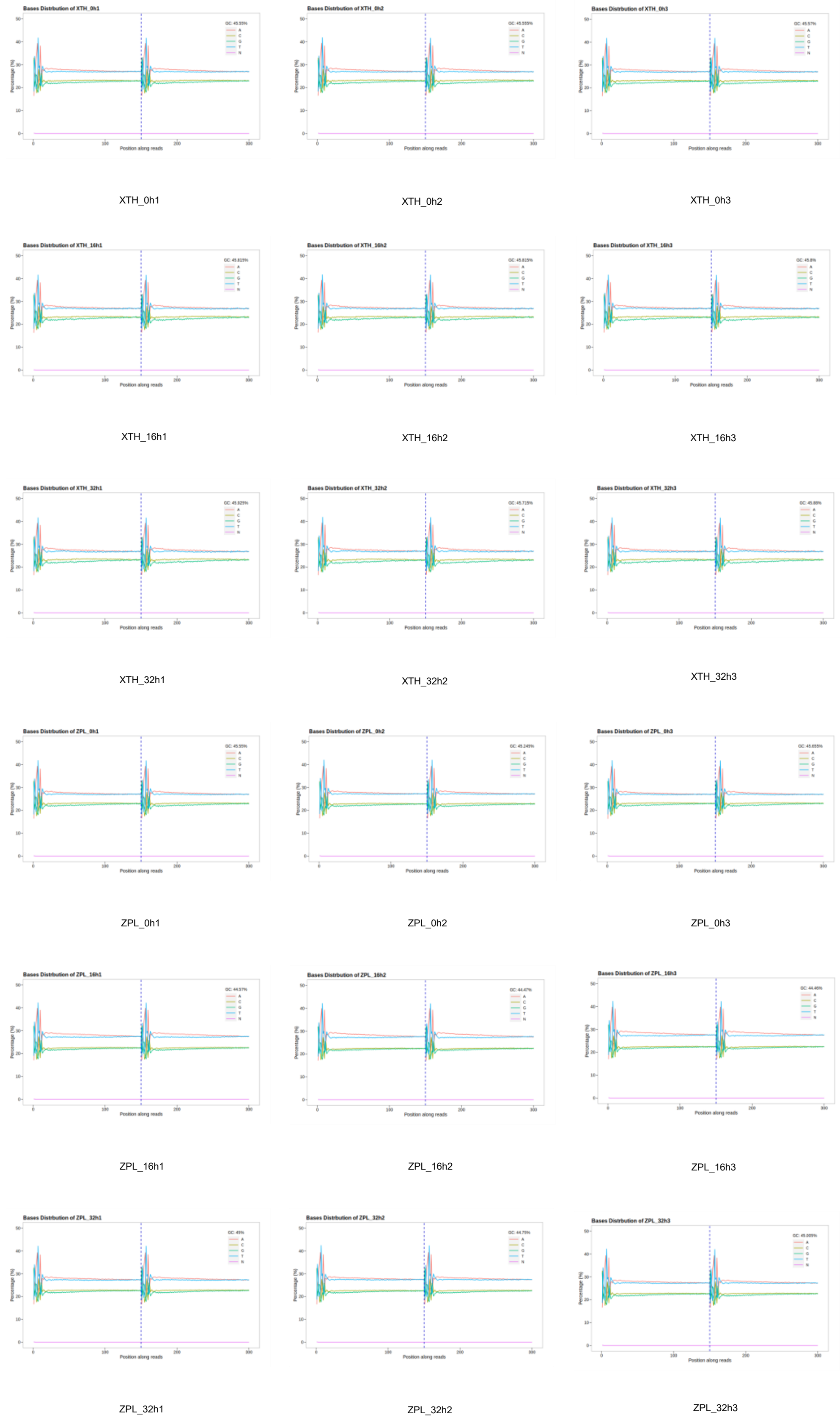

Figure S3. GC content of 18 RNA sequencing samples

Table S1. RNA-Seq data information of 18 RNA sequencing samples

Sample	Raw Reads	Clean Reads	Clean Base (G)	Error Rate (%)	Q20 (%)	Q30 (%)	GC Content (%)
XTH 0h1	55416920	52891068	7.93	0.02	98.51	95.1	45.55
XTH 0h2	52858684	51526524	7.73	0.02	98.32	94.59	45.55
XTH 0h3	52087826	49805826	7.47	0.02	98.48	95.01	45.57
XTH 16h1	65449210	62810770	9.42	0.02	98.45	94.88	45.81
XTH 16h2	57410358	55482486	8.32	0.02	98.41	94.82	45.81
XTH 16h3	54179448	52088102	7.81	0.02	98.49	95.05	45.8
XTH 32h1	57605166	55385420	8.31	0.02	98.53	95.15	45.92
XTH 32h2	53935430	52273264	7.84	0.02	98.38	94.75	45.71
XTH 32h3	57556094	55918352	8.39	0.02	98.52	95.14	45.88
ZPL 0h1	51145186	49061824	7.36	0.02	98.36	94.71	45.55
ZPL 0h2	53609208	51476214	7.72	0.02	98.35	94.66	45.24
ZPL 0h3	52634612	50852610	7.63	0.02	98.39	94.79	45.65
ZPL 16h1	51145058	49086616	7.36	0.02	98.36	94.73	44.57
ZPL 16h2	49359376	47080970	7.06	0.02	98.37	94.73	44.47
ZPL 16h3	59566366	57090130	8.56	0.02	98.37	94.76	44.46
ZPL 32h1	53596994	51523328	7.73	0.02	98.38	94.78	45
ZPL 32h2	53513156	51378254	7.71	0.02	98.34	94.66	44.75
ZPL 32h3	53900760	51163582	7.67	0.02	98.41	94.86	45
Average	54720547.33	52605296.67	7.89	0.02	98.41	94.82	45.35

Table S2. Statistics of length and number for the Trinity assembled transcripts and genes

Type	Number	Mean Length	N50	N90	Total Bases
Transcript	335764	880	1399	363	295475762
Unigene	197478	1116	1587	508	220440608

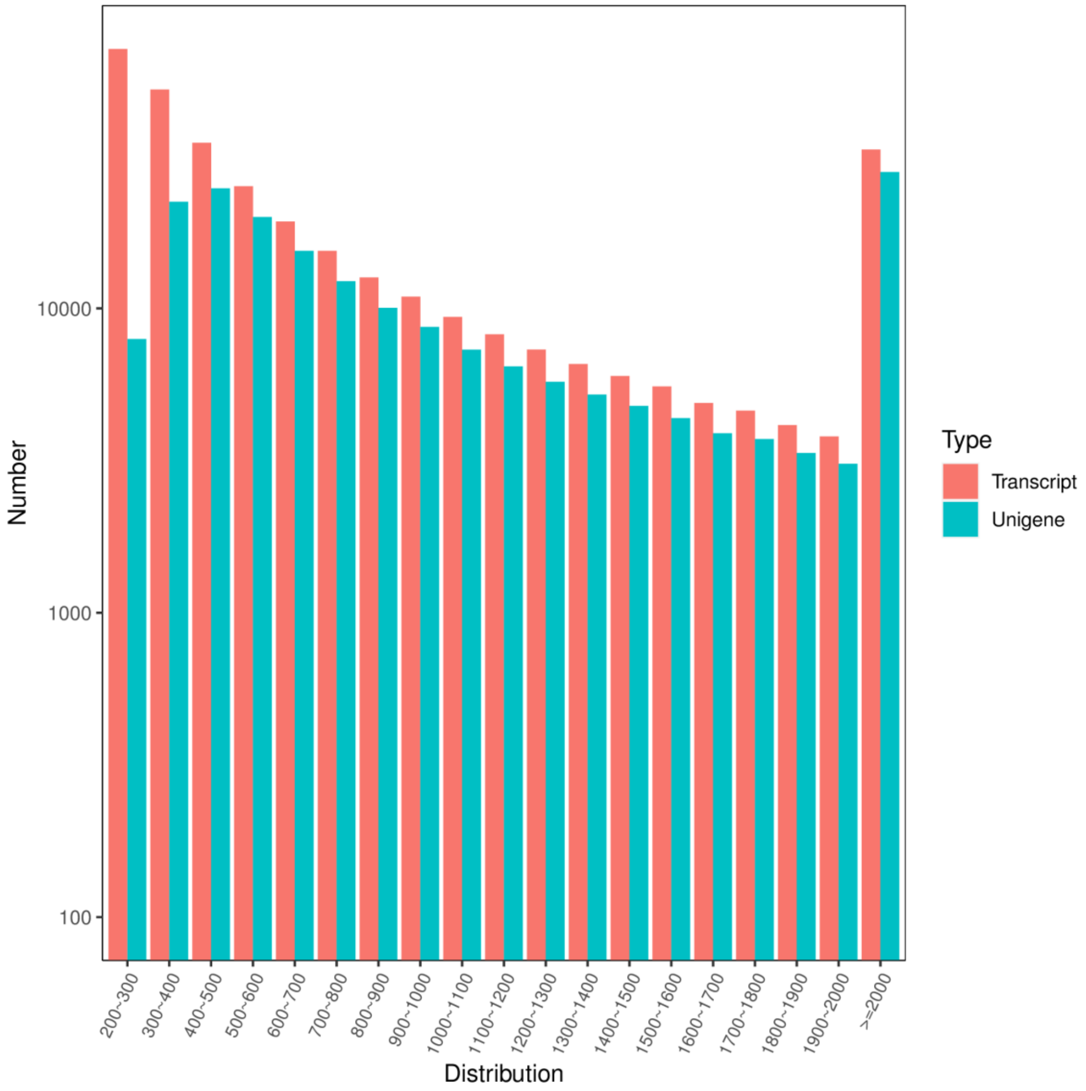

Figure S4. Quantity and average length of Trinity and Unigene obtained by splicing

Table S3. Unigenes were annotated information by database

Database	Number of Genes	Percentage （%）
KEGG	85436	43.26
Nr	143276	72.55
SwissProt	84846	42.96
TrEMBL	137994	69.88
KOG	71821	36.37
GO	114075	57.77
Pfam	71855	36.39
Annotated in at least one Database	146054	73.96
Total Unigenes	197478	100

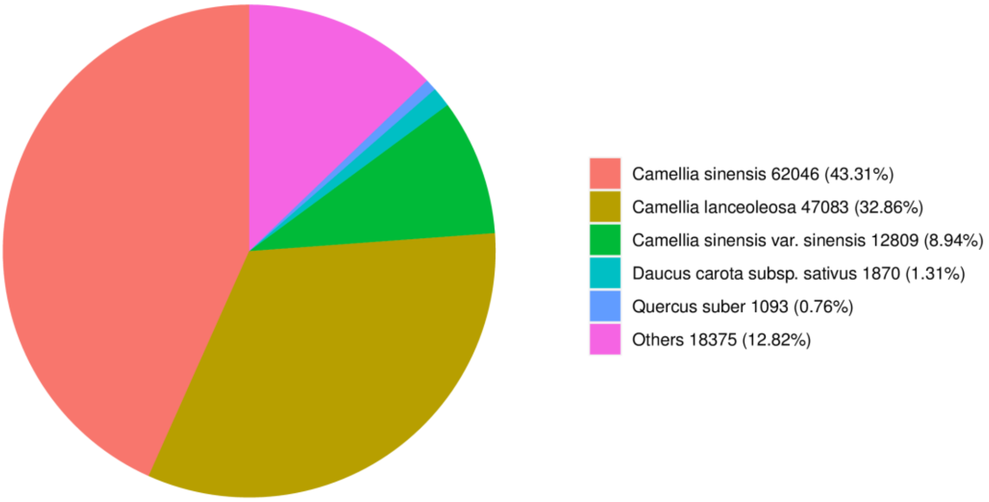

Figure S5. Unigene blast in NR database

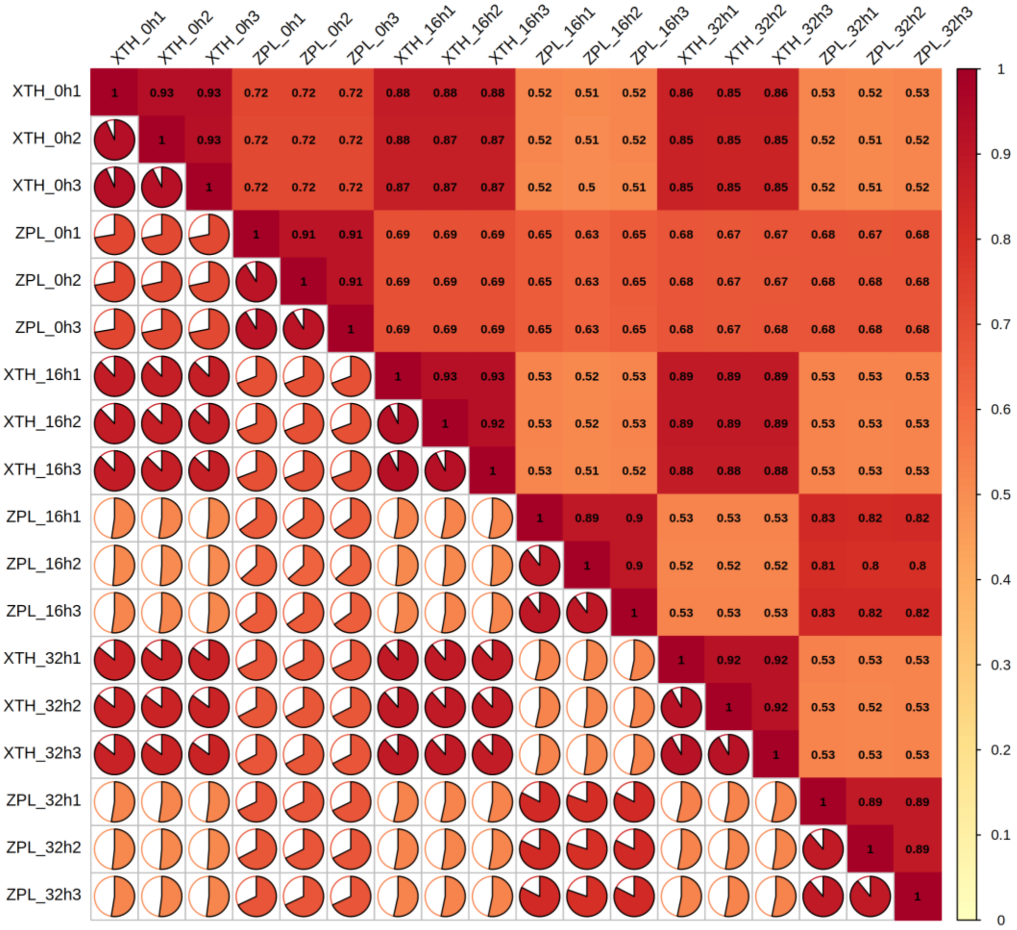

Figure S6. Pearson correlation coefficients of 18 samples

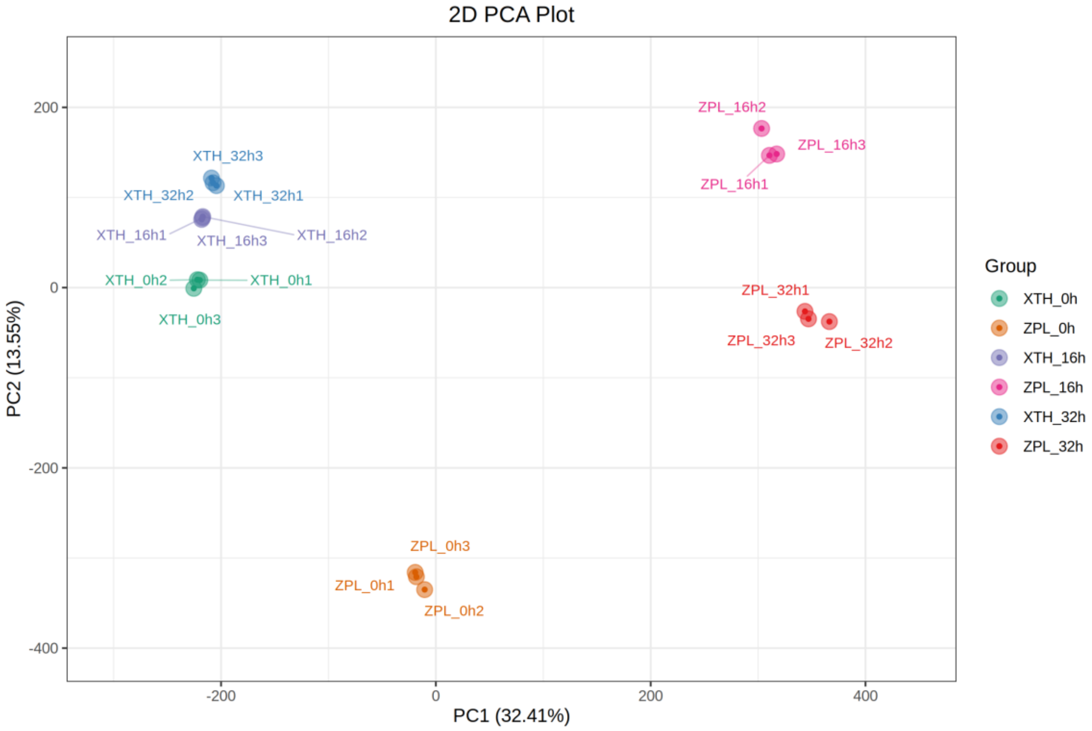

Figure S7. PCA analysis of 18 samples

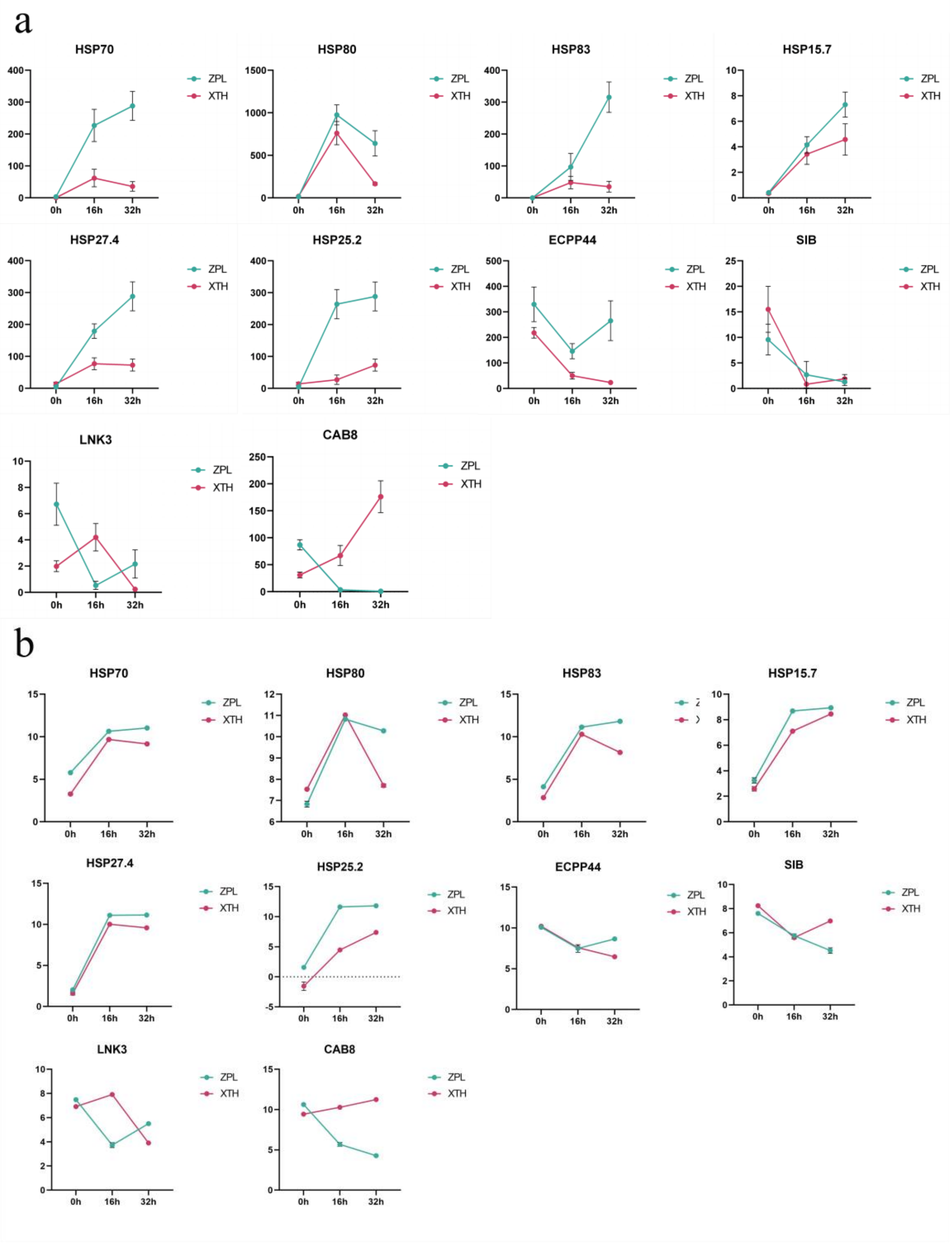

Figure S8. qRT-PCR analysis and expression trend results of 10 DEGs. (a) Result of 10 selected genes expression by qRT-PCR, calculated with $2^{-\Delta t}$; (b) Result of 10 selected genes expression in RNA-Seq, calculated with $\log_2$FPKM.

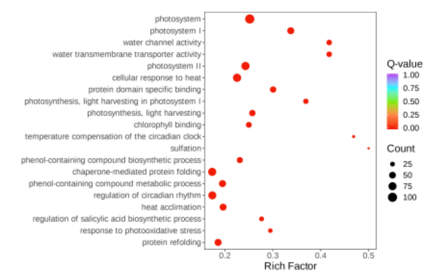

XTH_16h_vs_XTH_0h

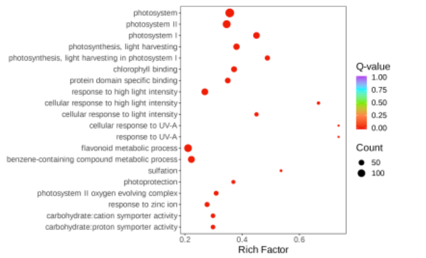

XTH_32h_vs_XTH_0h

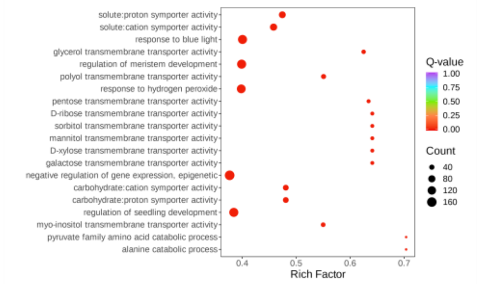

ZPL_16h_vs_ZPL_0h

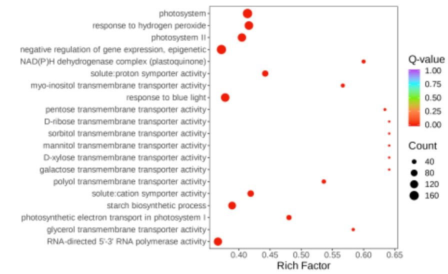

ZPL_32h_vs_ZPL_0h

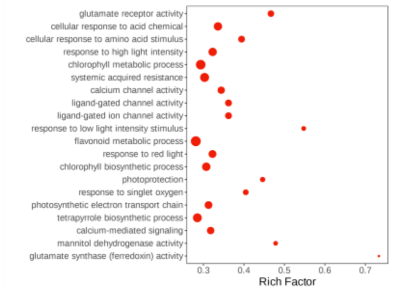

ZPL_0h_vs_XTH_0h

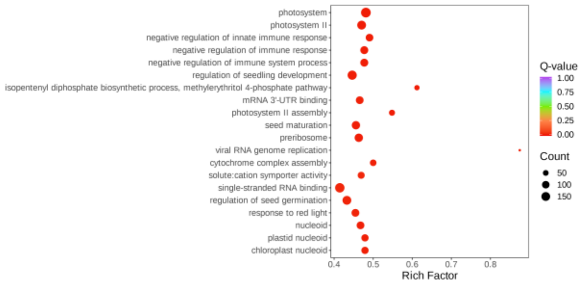

ZPL_16h_vs_XTH_16h

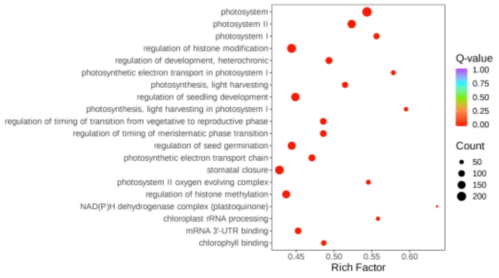

ZPL_32h_vs_XTH_32h

Figure S9. GO enrichment of DEGs

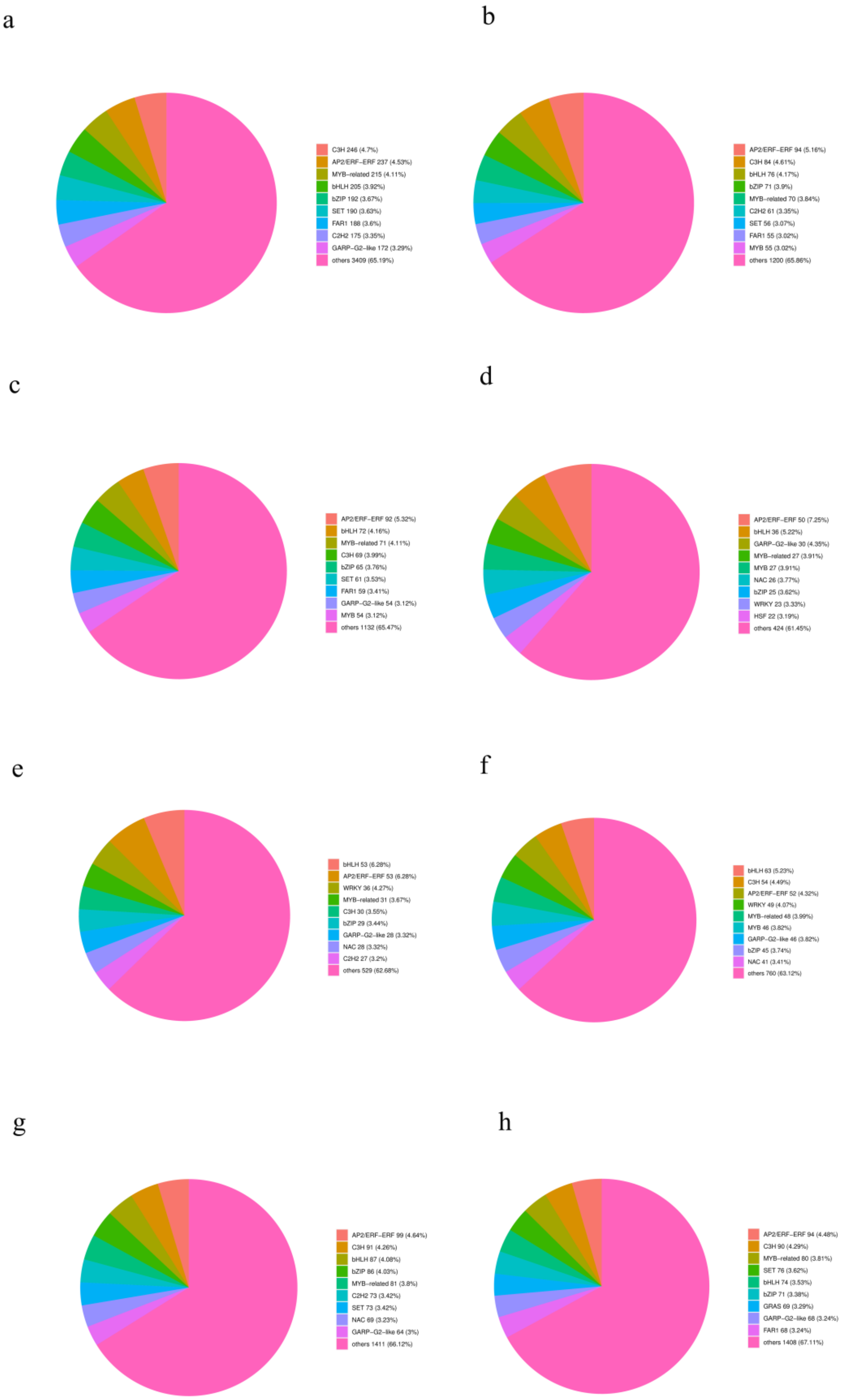

Figure S10. Pie chart of transcription factor annotation classification
(a) TFs in all DEGs; (b) DETFs of 'ZPL' in 16 h treatment; (c) DETFs of 'ZPL' in 32 h treatment; (d) DETFs of 'XTH' in 16 h treatment; (e) DETFs of 'XTH' in 32 h treatment; (f) DETFs betwe-en 'ZPL'and'XTH'in 0 h treatment; (g) DETFs between 'ZPL'and'XTH'in 16 h treatme-nt; (h) DETFs between 'ZPL'and'XTH'in 32 h treatment; QXH-'Qixinhong'; XTH-'Xiaotaohong'; ZPL-'Zhuapolian'; SBXS-'Shibaxueshi'

Table S4. Primer of qRT-PCR

Unigene ID	Unigene name	Forward primer sequence (5′-3′)	Reverse primer sequence (5′-3′)
Cluster-45777.0	*UBTD1*	GGAGACGGGACCATGAAGAA	CCCAGAACTCCTCACGCATC
Cluster-58070.36	*HSP70*	GGCGAAGCGAAGCAATTCTC	TTTCGTTGCCTGTCTCTGGG
Cluster-49234.7	*HSP80*	CTGCCACCAAGGAAGGTTTG	TCCAGTCACCAAACAGCAGG
Cluster-70892.0	*HSP83*	CTCACCATTTTTGGAGCGGC	AGTCACCAAACAGCAGGGAG
Cluster-94537.4	*HSP15.7*	TGAGAACCCGAACAACGCTT	ATTGGGGGTTTCAAGCCAGT
Cluster-88340.3	*HSP27.4*	CGCTCCCCATATCATCCCAAA	GGCTCTTGTTTGCTCTCTGC
Cluster-19537.1	*HSP25.2*	CTCAGGCCACCGGAGATAAC	GCCCTCATTGGAGACAGAGG
Cluster-79672.11	*ECPP44*	GCGGGCTCTTCGGTTTTATG	CTGCTATTGGATCGGGGGAG
Cluster-80133.1	*SIB*	AGAAGGCAACCAAACAGACCA	ACTTACCGTGTTCCGGCATC
Cluster-84032.0	*LNK3*	GAGGGTTCACTTCAGCAGGG	ATCAGCGGGCAATACACCAT
Cluster-80035.3	*CAB8*	GACCTCTCTGGTTTGCCTCC	CCTGTTTTGAACCACGGCAG
